# Supplementary material for: An open-source framework for large-scale, flexible evaluation of biomedical text mining systems
Source: J Biomed Discov Collab. 2008 Jan 29;3:1. doi: 10.1186/1747-5333-3-1 (PMC2276192; doi:10.1186/1747-5333-3-1)
Supplement: Additional file 1 — Evaluation platform source code. The additional file contains the software evaluation framework discussed in this paper. The most current release can be downloaded from SourceForge.net [1]. See the accompanying README file for instructions on installation and use. Also included in the distribution is a collection of UIMA wrappers for some commonly used BioNLP tools and annotated corpora. [file 1747-5333-3-1-S1.zip › bionlp_uima_repository-1.0.1/docs/api/edu/uchsc/ccp/util/nlp/mention/ClassMentionTypes.html]

ClassMentionTypes (Center for Computational Pharmacology Util/UIMA API)


|  |  |  |
| --- | --- | --- |
| **Overview** \| **Package** \| **Class** \| **Tree** \| **Deprecated** \| **Index** \| **Help** | |  |
| **PREV CLASS**   **NEXT CLASS** | **FRAMES**    **NO FRAMES**     **All Classes** |  |
| SUMMARY: NESTED \| FIELD \| CONSTR \| METHOD | DETAIL: FIELD \| CONSTR \| METHOD |  |


---


## edu.uchsc.ccp.util.nlp.mention Interface ClassMentionTypes

---

``` public interface ClassMentionTypes ```

---

| **Field Summary** | |
| --- | --- |
| `static String` | `ALANINE` |
| `static String` | `AMINO_ACID` |
| `static String` | `ARGININE` |
| `static String` | `ASPARAGINE` |
| `static String` | `ASPARTATE` |
| `static String` | `BIOLOGICAL_SEQUENCE` |
| `static String` | `BIOLOGICAL_SEQUENCE_ELEMENT` |
| `static String` | `BIOLOGICAL_SEQUENCE_POSITION` |
| `static String` | `BIOLOGICAL_SUBSEQUENCE` |
| `static String` | `CELL_LINE` |
| `static String` | `CELL_TYPE` |
| `static String` | `CLASS_MENTION_TYPE`             ClassMention Identifier - if this changes, Annotation DB must be adjusted accordingly |
| `static String` | `CLAUSE` |
| `static String` | `CYSTEINE` |
| `static String` | `DELETION` |
| `static String` | `DISEASE` |
| `static String` | `DNA` |
| `static String` | `DOCUMENT_FIGURE_LEGEND` |
| `static String` | `DOCUMENT_SECTION` |
| `static String` | `DOCUMENT_SECTION_HEADING` |
| `static String` | `DOCUMENT_SUBSECTION` |
| `static String` | `DOCUMENT_SUBSECTION_HEADING` |
| `static String` | `DOCUMENT_TABLE_LEGEND` |
| `static String` | `DOCUMENT_TITLE` |
| `static String` | `EXPRESSION`             Semantic predicates |
| `static String` | `GENE` |
| `static String` | `GENE_GENERIC` |
| `static String` | `GENE_OR_GENE_PRODUCT` |
| `static String` | `GENIA_atom` |
| `static String` | `GENIA_body_part` |
| `static String` | `GENIA_carbohydrate` |
| `static String` | `GENIA_cell_component` |
| `static String` | `GENIA_DNA_domain_or_region` |
| `static String` | `GENIA_DNA_family_or_group` |
| `static String` | `GENIA_DNA_NA` |
| `static String` | `GENIA_DNA_substructure` |
| `static String` | `GENIA_inorganic` |
| `static String` | `GENIA_lipid` |
| `static String` | `GENIA_mono_cell` |
| `static String` | `GENIA_multi_cell` |
| `static String` | `GENIA_nucleotide` |
| `static String` | `GENIA_other_artificial_source`             For GENIA tagger |
| `static String` | `GENIA_other_name` |
| `static String` | `GENIA_other_organic_compound` |
| `static String` | `GENIA_peptide` |
| `static String` | `GENIA_polynucleotide` |
| `static String` | `GENIA_protein_domain_or_region` |
| `static String` | `GENIA_protein_family_or_group` |
| `static String` | `GENIA_protein_NA` |
| `static String` | `GENIA_protein_substructure` |
| `static String` | `GENIA_protein_subunit` |
| `static String` | `GENIA_RNA_domain_or_region` |
| `static String` | `GENIA_RNA_family_or_group` |
| `static String` | `GENIA_RNA_NA` |
| `static String` | `GENIA_RNA_substructure` |
| `static String` | `GLUTAMATE` |
| `static String` | `GLUTAMINE` |
| `static String` | `GLYCINE` |
| `static String` | `HISTIDINE` |
| `static String` | `HTML_PARAGRAPH`             Document level Class Mentions |
| `static String` | `HTML_PARAGRAPH_END` |
| `static String` | `HTML_PARAGRAPH_START` |
| `static String` | `HTML_PARAGRAPH_TAG` |
| `static String` | `INSERTION` |
| `static String` | `ISOLEUCINE` |
| `static String` | `LEUCINE` |
| `static String` | `LYSINE` |
| `static String` | `MACROMOLECULE` |
| `static String` | `MALIGNANCY`             For Penn Bio Tagger |
| `static String` | `METHIONINE` |
| `static String` | `MUTATION_EVENT`             for Mutation project |
| `static String` | `NOMINALIZATION`             For Nominalization project |
| `static String` | `NOMINALIZATION_ACTIVATION` |
| `static String` | `NOMINALIZATION_DEVELOPMENT` |
| `static String` | `NOMINALIZATION_EXPRESSION` |
| `static String` | `NOMINALIZATION_FUNCTION` |
| `static String` | `NOMINALIZATION_GROWTH` |
| `static String` | `NOMINALIZATION_INHIBITION` |
| `static String` | `NOMINALIZATION_INTERACTION` |
| `static String` | `NOMINALIZATION_MUTATION` |
| `static String` | `NOMINALIZATION_PHOSPHORYLATION` |
| `static String` | `NOMINALIZATION_REGULATION` |
| `static String` | `NOMINALIZATION_RESPONSE` |
| `static String` | `NOMINALIZATION_TRANSCRIPTION` |
| `static String` | `NUCLEIC_ACID` |
| `static String` | `ORGAN`             for Olga's concept recognizers |
| `static String` | `ORGANISM` |
| `static String` | `PDG_ACTION` |
| `static String` | `PHENYLALANINE` |
| `static String` | `PHRASE` |
| `static String` | `POLYPEPTIDE_SEQUENCE` |
| `static String` | `POLYPEPTIDE_SUBSEQUENCE` |
| `static String` | `PROLINE` |
| `static String` | `PROTEIN`             Semantic Class Mentions |
| `static String` | `PROTEIN_ACRONYM` |
| `static String` | `PROTEIN_COMPLEX` |
| `static String` | `PROTEIN_COMPOUND` |
| `static String` | `PROTEIN_LONG_FORM` |
| `static String` | `RNA` |
| `static String` | `SENTENCE` |
| `static String` | `SERINE` |
| `static String` | `SUBCELLULAR_LOCATION` |
| `static String` | `SUBSTITUTION` |
| `static String` | `TAXON`             For Species Classification |
| `static String` | `THREONINE` |
| `static String` | `TISSUE` |
| `static String` | `TOKEN`             Syntactic Class Mentions |
| `static String` | `TRANSPORT_GATED` |
| `static String` | `TRYPTOPHAN` |
| `static String` | `TYROSINE` |
| `static String` | `UNDEFINED`             For MedLEE |
| `static String` | `VALINE` |
| `static String` | `VARIATION_EVENT` |
| `static String` | `VARIATION_TYPE` |
| `static String` | `VIRUS` |

**Field Detail**

### CLASS\_MENTION\_TYPE

```
static final String CLASS_MENTION_TYPE
```

:   ClassMention Identifier - if this changes, Annotation DB must be adjusted accordingly

    **See Also:**: Constant Field Values

---


### ORGAN

```
static final String ORGAN
```

:   for Olga's concept recognizers

    **See Also:**: Constant Field Values

---


### DISEASE

```
static final String DISEASE
```

**See Also:**: Constant Field Values

---


### HTML\_PARAGRAPH

```
static final String HTML_PARAGRAPH
```

:   Document level Class Mentions

    **See Also:**: Constant Field Values

---


### HTML\_PARAGRAPH\_START

```
static final String HTML_PARAGRAPH_START
```

**See Also:**: Constant Field Values

---


### HTML\_PARAGRAPH\_END

```
static final String HTML_PARAGRAPH_END
```

**See Also:**: Constant Field Values

---


### HTML\_PARAGRAPH\_TAG

```
static final String HTML_PARAGRAPH_TAG
```

**See Also:**: Constant Field Values

---


### DOCUMENT\_TITLE

```
static final String DOCUMENT_TITLE
```

**See Also:**: Constant Field Values

---


### DOCUMENT\_SECTION\_HEADING

```
static final String DOCUMENT_SECTION_HEADING
```

**See Also:**: Constant Field Values

---


### DOCUMENT\_SUBSECTION\_HEADING

```
static final String DOCUMENT_SUBSECTION_HEADING
```

**See Also:**: Constant Field Values

---


### DOCUMENT\_TABLE\_LEGEND

```
static final String DOCUMENT_TABLE_LEGEND
```

**See Also:**: Constant Field Values

---


### DOCUMENT\_FIGURE\_LEGEND

```
static final String DOCUMENT_FIGURE_LEGEND
```

**See Also:**: Constant Field Values

---


### TOKEN

```
static final String TOKEN
```

:   Syntactic Class Mentions

    **See Also:**: Constant Field Values

---


### PHRASE

```
static final String PHRASE
```

**See Also:**: Constant Field Values

---


### CLAUSE

```
static final String CLAUSE
```

**See Also:**: Constant Field Values

---


### SENTENCE

```
static final String SENTENCE
```

**See Also:**: Constant Field Values

---


### EXPRESSION

```
static final String EXPRESSION
```

:   Semantic predicates

    **See Also:**: Constant Field Values

---


### PROTEIN

```
static final String PROTEIN
```

:   Semantic Class Mentions

    **See Also:**: Constant Field Values

---


### GENE

```
static final String GENE
```

**See Also:**: Constant Field Values

---


### CELL\_TYPE

```
static final String CELL_TYPE
```

**See Also:**: Constant Field Values

---


### PDG\_ACTION

```
static final String PDG_ACTION
```

**See Also:**: Constant Field Values

---


### TRANSPORT\_GATED

```
static final String TRANSPORT_GATED
```

**See Also:**: Constant Field Values

---


### GENE\_OR\_GENE\_PRODUCT

```
static final String GENE_OR_GENE_PRODUCT
```

**See Also:**: Constant Field Values

---


### MACROMOLECULE

```
static final String MACROMOLECULE
```

**See Also:**: Constant Field Values

---


### NUCLEIC\_ACID

```
static final String NUCLEIC_ACID
```

**See Also:**: Constant Field Values

---


### CELL\_LINE

```
static final String CELL_LINE
```

**See Also:**: Constant Field Values

---


### VIRUS

```
static final String VIRUS
```

**See Also:**: Constant Field Values

---


### TISSUE

```
static final String TISSUE
```

**See Also:**: Constant Field Values

---


### SUBCELLULAR\_LOCATION

```
static final String SUBCELLULAR_LOCATION
```

**See Also:**: Constant Field Values

---


### ORGANISM

```
static final String ORGANISM
```

**See Also:**: Constant Field Values

---


### DNA

```
static final String DNA
```

**See Also:**: Constant Field Values

---


### RNA

```
static final String RNA
```

**See Also:**: Constant Field Values

---


### PROTEIN\_ACRONYM

```
static final String PROTEIN_ACRONYM
```

**See Also:**: Constant Field Values

---


### PROTEIN\_COMPOUND

```
static final String PROTEIN_COMPOUND
```

**See Also:**: Constant Field Values

---


### PROTEIN\_LONG\_FORM

```
static final String PROTEIN_LONG_FORM
```

**See Also:**: Constant Field Values

---


### GENE\_GENERIC

```
static final String GENE_GENERIC
```

**See Also:**: Constant Field Values

---


### MALIGNANCY

```
static final String MALIGNANCY
```

:   For Penn Bio Tagger

    **See Also:**: Constant Field Values

---


### VARIATION\_EVENT

```
static final String VARIATION_EVENT
```

**See Also:**: Constant Field Values

---


### VARIATION\_TYPE

```
static final String VARIATION_TYPE
```

**See Also:**: Constant Field Values

---


### GENIA\_other\_artificial\_source

```
static final String GENIA_other_artificial_source
```

:   For GENIA tagger

    **See Also:**: Constant Field Values

---


### PROTEIN\_COMPLEX

```
static final String PROTEIN_COMPLEX
```

**See Also:**: Constant Field Values

---


### GENIA\_other\_organic\_compound

```
static final String GENIA_other_organic_compound
```

**See Also:**: Constant Field Values

---


### GENIA\_RNA\_substructure

```
static final String GENIA_RNA_substructure
```

**See Also:**: Constant Field Values

---


### GENIA\_protein\_substructure

```
static final String GENIA_protein_substructure
```

**See Also:**: Constant Field Values

---


### GENIA\_carbohydrate

```
static final String GENIA_carbohydrate
```

**See Also:**: Constant Field Values

---


### GENIA\_nucleotide

```
static final String GENIA_nucleotide
```

**See Also:**: Constant Field Values

---


### GENIA\_peptide

```
static final String GENIA_peptide
```

**See Also:**: Constant Field Values

---


### GENIA\_multi\_cell

```
static final String GENIA_multi_cell
```

**See Also:**: Constant Field Values

---


### GENIA\_lipid

```
static final String GENIA_lipid
```

**See Also:**: Constant Field Values

---


### GENIA\_atom

```
static final String GENIA_atom
```

**See Also:**: Constant Field Values

---


### GENIA\_RNA\_NA

```
static final String GENIA_RNA_NA
```

**See Also:**: Constant Field Values

---


### GENIA\_body\_part

```
static final String GENIA_body_part
```

**See Also:**: Constant Field Values

---


### GENIA\_polynucleotide

```
static final String GENIA_polynucleotide
```

**See Also:**: Constant Field Values

---


### GENIA\_inorganic

```
static final String GENIA_inorganic
```

**See Also:**: Constant Field Values

---


### GENIA\_protein\_domain\_or\_region

```
static final String GENIA_protein_domain_or_region
```

**See Also:**: Constant Field Values

---


### GENIA\_DNA\_domain\_or\_region

```
static final String GENIA_DNA_domain_or_region
```

**See Also:**: Constant Field Values

---


### GENIA\_DNA\_substructure

```
static final String GENIA_DNA_substructure
```

**See Also:**: Constant Field Values

---


### GENIA\_protein\_family\_or\_group

```
static final String GENIA_protein_family_or_group
```

**See Also:**: Constant Field Values

---


### GENIA\_cell\_component

```
static final String GENIA_cell_component
```

**See Also:**: Constant Field Values

---


### GENIA\_RNA\_domain\_or\_region

```
static final String GENIA_RNA_domain_or_region
```

**See Also:**: Constant Field Values

---


### GENIA\_other\_name

```
static final String GENIA_other_name
```

**See Also:**: Constant Field Values

---


### GENIA\_DNA\_NA

```
static final String GENIA_DNA_NA
```

**See Also:**: Constant Field Values

---


### GENIA\_protein\_NA

```
static final String GENIA_protein_NA
```

**See Also:**: Constant Field Values

---


### GENIA\_protein\_subunit

```
static final String GENIA_protein_subunit
```

**See Also:**: Constant Field Values

---


### GENIA\_RNA\_family\_or\_group

```
static final String GENIA_RNA_family_or_group
```

**See Also:**: Constant Field Values

---


### GENIA\_DNA\_family\_or\_group

```
static final String GENIA_DNA_family_or_group
```

**See Also:**: Constant Field Values

---


### GENIA\_mono\_cell

```
static final String GENIA_mono_cell
```

**See Also:**: Constant Field Values

---


### MUTATION\_EVENT

```
static final String MUTATION_EVENT
```

:   for Mutation project

    **See Also:**: Constant Field Values

---


### SUBSTITUTION

```
static final String SUBSTITUTION
```

**See Also:**: Constant Field Values

---


### DELETION

```
static final String DELETION
```

**See Also:**: Constant Field Values

---


### INSERTION

```
static final String INSERTION
```

**See Also:**: Constant Field Values

---


### BIOLOGICAL\_SEQUENCE

```
static final String BIOLOGICAL_SEQUENCE
```

**See Also:**: Constant Field Values

---


### POLYPEPTIDE\_SEQUENCE

```
static final String POLYPEPTIDE_SEQUENCE
```

**See Also:**: Constant Field Values

---


### BIOLOGICAL\_SEQUENCE\_POSITION

```
static final String BIOLOGICAL_SEQUENCE_POSITION
```

**See Also:**: Constant Field Values

---


### BIOLOGICAL\_SEQUENCE\_ELEMENT

```
static final String BIOLOGICAL_SEQUENCE_ELEMENT
```

**See Also:**: Constant Field Values

---


### AMINO\_ACID

```
static final String AMINO_ACID
```

**See Also:**: Constant Field Values

---


### ALANINE

```
static final String ALANINE
```

**See Also:**: Constant Field Values

---


### GLYCINE

```
static final String GLYCINE
```

**See Also:**: Constant Field Values

---


### VALINE

```
static final String VALINE
```

**See Also:**: Constant Field Values

---


### LEUCINE

```
static final String LEUCINE
```

**See Also:**: Constant Field Values

---


### ISOLEUCINE

```
static final String ISOLEUCINE
```

**See Also:**: Constant Field Values

---


### METHIONINE

```
static final String METHIONINE
```

**See Also:**: Constant Field Values

---


### PHENYLALANINE

```
static final String PHENYLALANINE
```

**See Also:**: Constant Field Values

---


### TYROSINE

```
static final String TYROSINE
```

**See Also:**: Constant Field Values

---


### TRYPTOPHAN

```
static final String TRYPTOPHAN
```

**See Also:**: Constant Field Values

---


### SERINE

```
static final String SERINE
```

**See Also:**: Constant Field Values

---


### PROLINE

```
static final String PROLINE
```

**See Also:**: Constant Field Values

---


### THREONINE

```
static final String THREONINE
```

**See Also:**: Constant Field Values

---


### CYSTEINE

```
static final String CYSTEINE
```

**See Also:**: Constant Field Values

---


### ASPARAGINE

```
static final String ASPARAGINE
```

**See Also:**: Constant Field Values

---


### GLUTAMINE

```
static final String GLUTAMINE
```

**See Also:**: Constant Field Values

---


### LYSINE

```
static final String LYSINE
```

**See Also:**: Constant Field Values

---


### HISTIDINE

```
static final String HISTIDINE
```

**See Also:**: Constant Field Values

---


### ARGININE

```
static final String ARGININE
```

**See Also:**: Constant Field Values

---


### ASPARTATE

```
static final String ASPARTATE
```

**See Also:**: Constant Field Values

---


### GLUTAMATE

```
static final String GLUTAMATE
```

**See Also:**: Constant Field Values

---


### BIOLOGICAL\_SUBSEQUENCE

```
static final String BIOLOGICAL_SUBSEQUENCE
```

**See Also:**: Constant Field Values

---


### POLYPEPTIDE\_SUBSEQUENCE

```
static final String POLYPEPTIDE_SUBSEQUENCE
```

**See Also:**: Constant Field Values

---


### NOMINALIZATION

```
static final String NOMINALIZATION
```

:   For Nominalization project

    **See Also:**: Constant Field Values

---


### NOMINALIZATION\_PHOSPHORYLATION

```
static final String NOMINALIZATION_PHOSPHORYLATION
```

**See Also:**: Constant Field Values

---


### NOMINALIZATION\_MUTATION

```
static final String NOMINALIZATION_MUTATION
```

**See Also:**: Constant Field Values

---


### NOMINALIZATION\_FUNCTION

```
static final String NOMINALIZATION_FUNCTION
```

**See Also:**: Constant Field Values

---


### NOMINALIZATION\_RESPONSE

```
static final String NOMINALIZATION_RESPONSE
```

**See Also:**: Constant Field Values

---


### NOMINALIZATION\_REGULATION

```
static final String NOMINALIZATION_REGULATION
```

**See Also:**: Constant Field Values

---


### NOMINALIZATION\_DEVELOPMENT

```
static final String NOMINALIZATION_DEVELOPMENT
```

**See Also:**: Constant Field Values

---


### NOMINALIZATION\_GROWTH

```
static final String NOMINALIZATION_GROWTH
```

**See Also:**: Constant Field Values

---


### NOMINALIZATION\_TRANSCRIPTION

```
static final String NOMINALIZATION_TRANSCRIPTION
```

**See Also:**: Constant Field Values

---


### NOMINALIZATION\_ACTIVATION

```
static final String NOMINALIZATION_ACTIVATION
```

**See Also:**: Constant Field Values

---


### NOMINALIZATION\_INTERACTION

```
static final String NOMINALIZATION_INTERACTION
```

**See Also:**: Constant Field Values

---


### NOMINALIZATION\_EXPRESSION

```
static final String NOMINALIZATION_EXPRESSION
```

**See Also:**: Constant Field Values

---


### NOMINALIZATION\_INHIBITION

```
static final String NOMINALIZATION_INHIBITION
```

**See Also:**: Constant Field Values

---


### UNDEFINED

```
static final String UNDEFINED
```

:   For MedLEE

    **See Also:**: Constant Field Values

---


### DOCUMENT\_SECTION

```
static final String DOCUMENT_SECTION
```

**See Also:**: Constant Field Values

---


### DOCUMENT\_SUBSECTION

```
static final String DOCUMENT_SUBSECTION
```

**See Also:**: Constant Field Values

---


### TAXON

```
static final String TAXON
```

:   For Species Classification

    **See Also:**: Constant Field Values


---


|  |  |  |
| --- | --- | --- |
| **Overview** \| **Package** \| **Class** \| **Tree** \| **Deprecated** \| **Index** \| **Help** | |  |
| **PREV CLASS**   **NEXT CLASS** | **FRAMES**    **NO FRAMES**     **All Classes** |  |
| SUMMARY: NESTED \| FIELD \| CONSTR \| METHOD | DETAIL: FIELD \| CONSTR \| METHOD |  |


---
